# The Application Status of Nanoscale Cellulose-Based Hydrogels in Tissue Engineering and Regenerative Biomedicine

**DOI:** 10.3389/fbioe.2021.732513

**Published:** 2021-11-18

**Authors:** Chenyang Wang, Jin Bai, Pei Tian, Rui Xie, Zifan Duan, Qinqin Lv, Yuqiang Tao

**Affiliations:** ^1^ School of Chemistry and Chemical Engineering, University of South China, Hengyang, China; ^2^ The Fourth College of Clinical Medicine, Zhejiang Chinese Medical University, Hangzhou, China

**Keywords:** nanoscale, cellulose, hydrogel, biomedicine, non-toxic material

## Abstract

As a renewable, biodegradable, and non-toxic material with moderate mechanical and thermal properties, nanocellulose-based hydrogels are receiving immense consideration for various biomedical applications. With the unique properties of excellent skeletal structure (hydrophilic functional groups) and micro-nano size (small size effect), nanocellulose can maintain the three-dimensional structure of the hydrogel to a large extent, providing mechanical strength while ensuring the moisture content. Owing to its unique features, nanocellulose-based hydrogels have made excellent progress in research and development on tissue engineering, drug carriers, wound dressings, development of synthetic organs, 3D printing, and biosensing. This review provides an overview of the synthesis of different types of nanocellulose, including cellulose nanocrystals, cellulose nanofibers, and bacterial nanocellulose, and describes their unique features. It further provides an updated knowledge of the development of nanocellulose-based functional biomaterials for various biomedical applications. Finally, it discusses the future perspective of nanocellulose-based research for its advanced biomedical applications.

## Introduction

**FIGURE 1:**
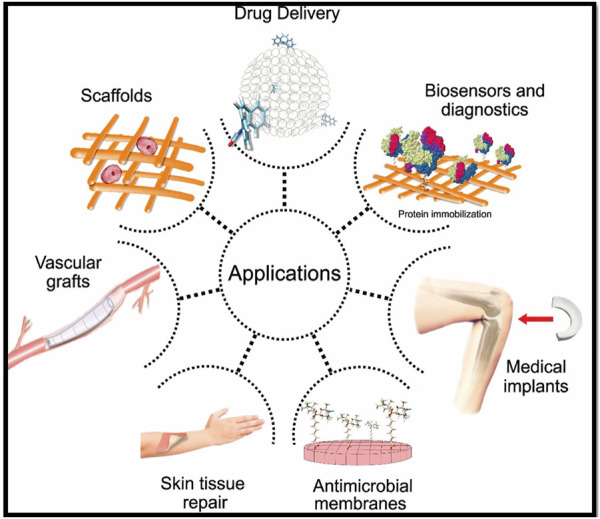
Biomedical applications of nanoscale cellulose hydrogels. Figure reproduced from Toro et al. (2021) under Creative Commons Attribution (CC BY) license.

Hydrogels are three-dimensional (3D) network materials consisting of cross-linked hydrophilic polymers. These have high and reversible uptake and release capability for water and other fluids (Shi et al., 2016; Arafiles and Futaki, 2021). Among the different polymer-based hydrogels, nanocellulose-based hydrogels are receiving immense consideration owing to their unique surface chemistry, high water holding capacity, moldability, flexibility, and biocompatibility. The cellulose-based hydrogels are widely used for different biomedical applications such as wound dressings (Sultan et al., 2017; Loh et al., 2018; Mao et al., 2021; Wang et al., 2021), tissue engineering (Boyer et al., 2018; Khan et al., 2021), drug delivery (Plackett et al., 2014; Hujaya et al., 2018; Loh et al., 2018), biosensing (Farooq et al., 2020; Subhedar et al., 2021), additive manufacturing (McCarthy et al., 2019a), food packaging (Atta et al., 2021), and several others (Ullah et al., 2021). Some biomedical applications of nanoscale cellulose hydrogels are summarized in Figure 1.

Hydrogels are broadly prepared by two methods: physical cross-linking and chemical cross-linking. The preparation of cellulose-based hydrogels through physical cross-linking takes advantage of the polyhydroxy nature of cellulose, forming many hydrogen bonding to support the mechanical stability of nanoscale cellulose-based hydrogels and form intertwined molecular chains to stabilize the 3D structure (Fatima et al., 2021; Ul-Islam et al., 2021). The physical cross-linked cellulose-based hydrogels are widely used in the preparation of biomedical materials because no other chemical substances are added to them, which may otherwise cause toxicity to the cells or tissues, and thus ensuring good biocompatibility without compromising the basic morphology and chemical properties. In contrast, the chemical cross-linked cellulose hydrogels are prepared through a chemical reaction between a compound and cellulose. The chemically crosslinked hydrogels demonstrate high mechanical strength and good elasticity. Moreover, these possess better wear resistance and service life than the physically crosslinked hydrogels.

Cellulose is the most abundant natural polymer on Earth. Its molecular skeleton contains numerous hydrophilic groups such as hydroxyl, aldehyde, and carboxyl groups. Its hydrophilic nature allows the formation of intra and inter-molecular hydrogen bonding, which imparts stability to its structure (France et al., 2017; Hujaya et al., 2018). Furthermore, its high aspect ratio and surface area allow its interaction with other materials of different nature such as polymers, nanoparticles, clays, and others (Shah et al., 2013; Khan et al., 2015a; Ullah et al., 2016a; Sajjad et al., 2019). Its high elastic modulus, high degree of surface functionalization, low density, good biocompatibility, and other properties of nanocellulose make it a material of choice for developing a variety of composite materials for different applications. Different types of nanocellulose not only serve as the matrix but also serve as the fillers or modifiers and enhance the morphological, mechanical, and thermal properties of the host materials.

In general, there are three classes of nanocellulose: cellulose nanocrystals (CNCs), cellulose nanofibers (CNFs), and bacterial nanocellulose (BNC). Among them, CNCs have become an excellent choice for surface modification and doping due to their high purity and high crystallinity, which can provide a rich composite space for the research and development of characteristic hydrogels when applied to the preparation of hydrogels. Similarly, CNFs due to their high degree of polymerization, high water absorption capacity, and high flexibility, can impart high mechanical strength tensile elasticity to hydrogels. Compared to CNCs and CNFs, the BNC has become the preferred material for the preparation of hydrogels due to its unique fibrous and network morphology similar to natural extracellular matrix (ECM), non-toxicity, biodegradability, high mechanical strength, flexibility, and moldability. The presence of rich intra and inter-molecular and intra-molecular hydrogen bonding stabilizes its structure which in turn enhances the mechanical strength of nanocellulose-based hydrogels (Yang et al., 2014). Due to its ability to main a dynamic balance of water content under the adjustment of osmotic pressure, BNC-based hydrogels are widely used in wound dressing where these prevent the pain and scarring when removing the wound dressing after recovery of wounds or when changing the bandage (Arfin, 2020).

This review provides a comprehensive overview of the preparation, properties, and biomedical applications of different types of nanocellulose-based hydrogels. In comparison to other reviews which mainly summarize the preparation of different types of nanocellulose and their biomedical applications, this review specifically focusses on the preparation of nanocellulose-based hydrogels, highlights the important features of nanocellulose as hydrogels and describes their structure-functional relationship, and finally summarizes the recent trends of applications in tissue engineering and regenerative medicine. We also discussed some future research directions for potential perspectives biomedical applications of nanocellulose.

## Types of Nanocellulose and Synthesis

**FIGURE 2:**
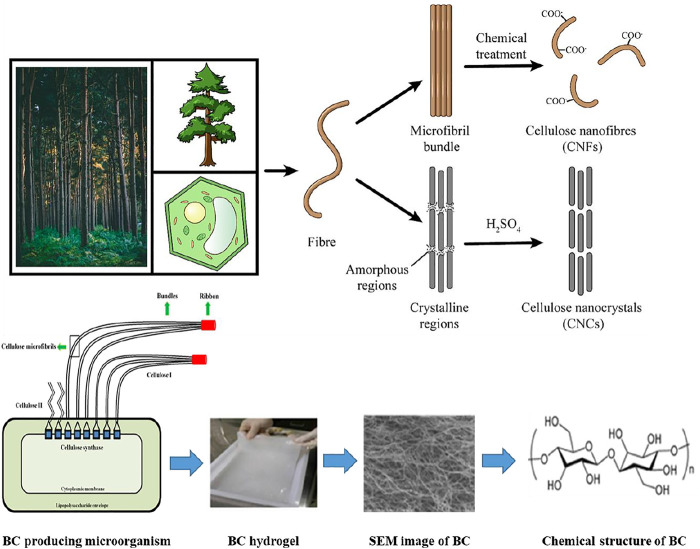
Preparations for three types of nanocellulose.

The three main classes of nanocellulose, CNCs, CNFs, and BNC, are differentiated from each other in terms of their synthesis method, morphology, and other properties. The following sections describe the synthesis of different types of nanocellulose and their characteristic properties. Table 1 provides a comparative analysis of the synthesis, morphology, dimension, and properties of different types of nanocellulose, while the synthesis of different types of nanocellulose is illustrated in Figure 2.

**TABLE 1:** A comparative analysis of synthesis, morphology, dimension, and properties of different types of nanocellulose.

Cellulose type	Synthesis method	Morphology and dimension	Properties	Reference
Cellulose nanocrystals	Acid hydrolysis	Rod/needle-shaped	High surface area (400–700 m^2^/g), high tensile strength (7,500 MPa), high stiffness (E > 140 GPa), and high aspect ratio (∼72), cellulose-I polymorphic structure	(France et al., 2017; Grishkewich et al., 2017; SD et al., 2019; Arfin, 2020)
		Length 150–300 nm		
Cellulose nanofibers	Physical shearing and homogenization	Mixture of amorphous and crystalline cellulose chains, 10–50 nm in diameter and 500–1,500 nm in length	High surface area (100 m^2^/g), high aspect ratio (∼100), great stiffness (E > 100 GPa), moderate crystallinity (<70%), cellulose-I polymorphic structure	(Nechyporchuk et al., 2016; Nascimento et al., 2018; Mohammad et al., 2020)
TEMPO-oxidized cellulose nanofibers	TEMPO (2,2,6,6-tetramethylpiperidine-1-oxyl radical)-mediated oxidation	Nanofibers of 3–4 nm diameter a few microns in length	Aspect ratios >100, cellulose-I polymorphic structure	Isogai and Bergström, (2018)
Bacterial nanocellulose	*In-vivo* synthesis in bacterial cells and extracellular organization into highly-ordered structures	Fiber diameter (5–10 nm), fiber length (70–80 nm), particle length (>1 µm), particle width (30–50 nm), particle height (6–10 nm)	High purity, high density (1.5 cm^−3^), high crystallinity (65–90%), high degree of polymerization (800–10,000), high surface area (24–40), highly porous, cellulose-I polymorphic structure	(Klemm et al., 2005; Ul-Islam et al., 2012, 2019a; Mishra et al., 2018; Ullah et al., 2019b)
Cell-free nanocellulose	*In-vitro* cell-free enzyme system	Nanofibers of 85–98 nm diameter and few microns in length	High water holding capacity (188.6 times of dry weight), high tensile strength (17.63 MPa), high thermal stability, cellulose-II polymorphic structure	(Ullah et al., 2015, 2017; Kim et al., 2019)

### Cellulose Nanocrystals

Cellulose nanocrystals or cellulose nanowhiskers are obtained from the amorphous sections of the wood pulp through acid hydrolysis (Trache et al., 2017). Acid hydrolysis is a two-step process: In the first step, alkaline treatment of wood pulp is carried out for the removal of polysaccharides at the fibril surface. In the second step, heat treatment in an acidic environment is carried out for cleaving and destructing the accessible amorphous regions of cellulose fibers. This usually involves the degradation of the amorphous region of cellulose to obtain a suspension of CNCs with high crystallinity. CNCs appear as rod or needle-like structures under scanning or transmission electron microscope. The structural and morphological features of CNCs are greatly dependent on the raw material, type of acid, reaction temperature and time, and intensity of ultrasonic irradiation (Chen et al., 2009). For example, changing the cellulose source from cotton fibers to ascidian produced CNCs of diameter 200–300 nm and >1 μm, respectively, under the same experimental conditions (Heux et al., 2000; Pakzad et al., 2012). The degree of hydrolysis of cellulose by inorganic acid is not only related to acid concentration but also related to the temperature. With the increase of hydrolysis temperature, the crystallinity of fiber first increases and then decreases, and there is a maximum value. The possible reason is that the aggregation ability of fiber increases with the increasing temperature and result in the decrease of crystallinity. A recent study reported the optimum parameters for sulfuric acid hydrolysis with acid hydrolysis time of 60 min, hydrolysis temperature of 45°C, sulfuric acid concentration of 64%, ultrasonic wave treatment for 30 min. Under such treatment, CNCs with a particle size of 18–26 nm were obtained (Tuerxun et al., 2019). In addition to acid hydrolysis, CNCs could also be obtained by treating the lignocellulosic material with reagents like tetramethyl-piperidine-1-oxyl (TEMPO) (Saito et al., 2007), ammonium persulfate (APS) (Wågberg et al., 2008), and some bio-based enzymes (Pääkko et al., 2007). Similarly, TEMPO-oxidized CNCs could be obtained by treating the lignocellulosic material with TEMPO, NaBr, and NaClO in an alkaline environment (Saito et al., 2007). Besides, CNCs could also be obtained through enzymatic hydrolysis of microcrystalline cellulose (Satyamurthy et al., 2011); however, this method is not feasible due to the high cost of enzymes.

### Cellulose Nanofibers

Cellulose nanofibers are obtained through grinding, homogenization, and ultrasonication of wood, cotton, fibers, and tunicate, etc. Among these methods, homogenization is commonly used; however, the traditional high-pressure homogenization has a long working cycle, low yield, and is easy to be blocked by cellulose. Besides, CNFs are also produced chemically, such as through oxidation of raw material by TEMPO under magnetic stirring (Saito et al., 2007). Sometimes, both physical and chemical processes are used together for the preparation of CNFs. For example, carboxymethylation and high-pressure homogenization produce uniformly distributed CNFs (Wågberg et al., 2008). The CNFs hydrogels are prepared through mechanical treatment of their aqueous suspension with alkali and neutralization (Abe and Yano, 2011, 2012). CNFs contain both amorphous and crystalline regions and are long and flexible interconnected fibrils. Similar to CNCs, their length and diameter also vary according to the type of source material. In general, CNFs vary in the diameter of 10–50 nm and length of 500–1,500 nm (Chen W. et al., 2018). These possess properties like high aspect ratio and surface area, high mechanical strength and crystallinity, and pre-colation threshold.

### Bacterial Nanocellulose

Bacterial nanocellulose is produced by a special class of acetic acid bacteria as well as by the cell-free enzyme systems. Compared to CNCs and CNFs, BNC represents the purest form of cellulose as it does not contain ingredients like hemicellulose, lignin, and minerals. The biosynthesis of BNC by these two systems is a chemical process mediated by specific enzymes. It possesses superior structural, physico-chemical, mechanical, and biological properties (Ul-Islam et al., 2019a). It is synthesized intracellularly by the microbial cells in the form of β-1,4-glucan chains. The chains are extruded to the external medium as protofibrils through the terminal complexes (TCs). The TCs are present in the outer cellular membrane. The excreted fibrils crystallize and form the ribbon-shaped microfibrils, which then form pellicles. The pellicles grow in size at the top of the air-medium interface. The pellicles are comprised of bundles, which then form the ribbon. The cellulose synthesis by microbial cells and cell-free enzyme system differs at the excretion step as the latter does not possess the external membrane barrier. Furthermore, the microbial cells produce cellulose I, while the cell-free enzyme system produces cellulose II polymorphic form (Ullah et al., 2016b; Kim et al., 2019).

## Biomedical Application of Nanocellulose

Different types of nanocellulose are receiving immense consideration for a range of applications in different fields, with particular attention received in the biomedical sector. The following sections overview the trends of biomedical applications of nanocellulose.

### Tissue Engineering Hydrogels and Scaffolds

Tissue engineering refers to the utilization of principles and methods of engineering and life science to fundamentally understand the structure-function relationship between the normal and diseases tissues with the aim to develop biological substitutes of damaged or lost organs and tissues and for restoring their lost function (Aljohani et al., 2018). Over the last few decades, this field has received immense consideration in biomedical research, and the potential of different materials has been widely explored to make scaffolds mimicking the natural tissues and organs. The tissue engineering scaffolds support the growth of cells by providing an appropriate environment for their adhesion, proliferation, and differentiation by providing nutrients and growth factors and allowing the exchange of metabolites and gases (Halib et al., 2019). Moreover, such scaffolds demonstrate features such as biocompatibility, non-toxicity, biodegradability, mechanical strength, plasticity, porosity, and others, which are modulated in the desired way by using the different combinations of natural and synthetic materials to meet the pre-requisites of the target tissue or organ (Moroni et al., 2014; Tummala, et al., 2019).

**FIGURE 3:**
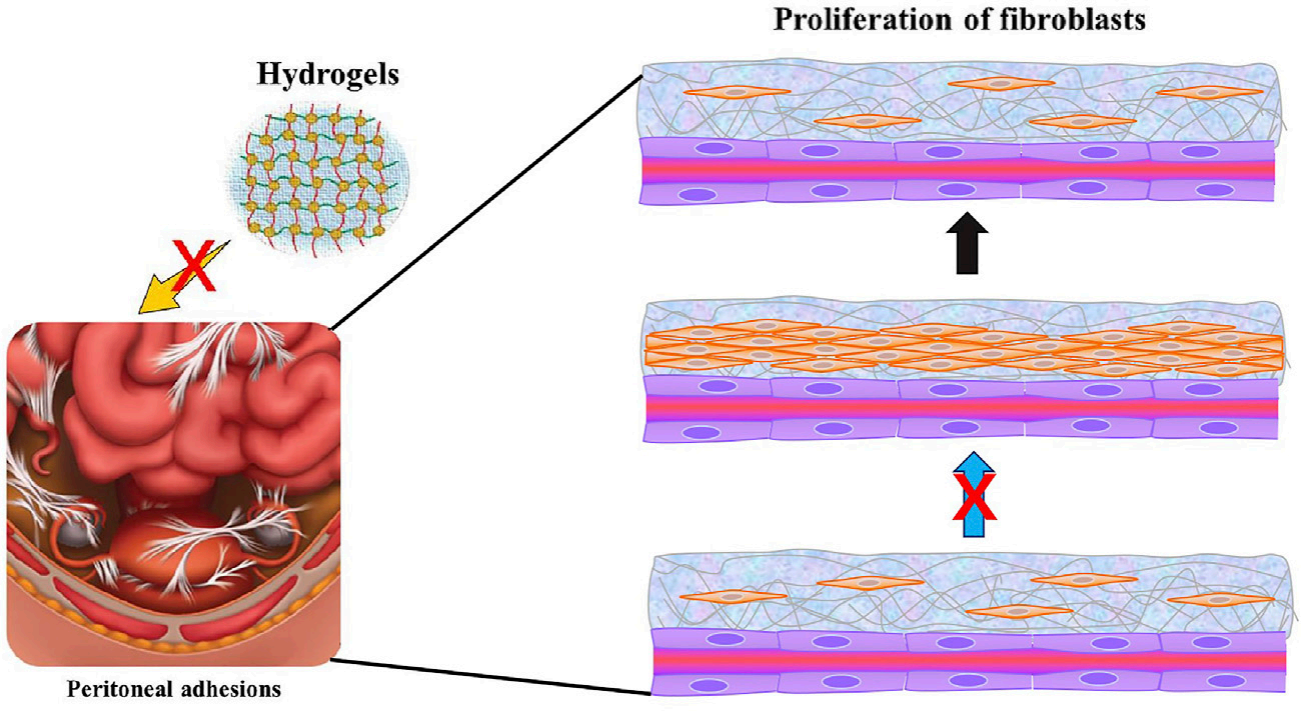
Schematic diagram of injectable anti-adhesion hydrogel.

Nanocellulose is considered an ideal material for the development of tissue engineering scaffold because of its renewability, biocompatibility, biodegradability, non-toxicity, rheological properties, and high mechanical strength (Lin and Dufresne, 2014). Over the last couple of decades, the hydrogels based on CNFs, CNCs, and BNC have been widely used in tissue engineering due to their highly hydrated porous 3D structure and excellent mechanical properties. Moreover, its gel-like and flexible 3D porous structure supports the adhesion and growth of cells and replicates the niche found *in vivo* (Curvello et al., 2019). The porous and 3D fibrous structure of BNC allows the gaseous and nutrients exchange. Most importantly, the nanocellulose-based hydrogels have structural similarity with the ECM enabling them to support the proliferation and differentiation of cells and thus making it a useful material for *in vitro* and *in vivo* applications. In a study, Sultana et al. developed heat-responsive TEMPO-oxidized nanocellulose (TOCN) injectable hydrogels for relieving the post-surgical peritoneal adhesion after surgery. The prepared physical barrier remains in a liquid state at low temperature (4°C) and self-transformed into gel at near human body temperature (37°C) in merely 45 s. After 14 days of experimental culture *in vitro*, the survival rates of 0.2% TOCN hydrogel on rat bone marrow mesenchymal stem cells (RBMSCs) and L929 fibroblast models were 89.24 and 91.25%, respectively, indicating the non-toxic nature of the developed hydrogel. In the rate cecal wall abrasion model, the developed hydrogel showed a good anti-peritoneal adhesion effect and effectively reduced the proliferation of fibroblasts. The developed hydrogel system effectively reduced post-surgery tissue adhesion. The injected TOCN hydrogel as a sol-gel barrier can be used under laparoscope and thus could potentially avoid the second surgery after injury (Sultana et al., 2019). The nanocellulose-based hydrogel system can be a cost-effective and efficient substitute for commercial hydrogels owing to the renewability, biocompatibility, and biodegradability of nanocellulose. The development and application of heat-sensitive nanocellulose-based injectable hydrogel are illustrated in Figure 3. Furthermore, Table 2 summarizes the development of different nanocellulose-based scaffolds with a variety of materials for different tissue engineering applications.

**TABLE 2:** The improved properties and targets of different nanocellulose-based tissue engineering scaffolds.

Cellulose type	Reinforcement material	Synthetic strategy	Improved/Added properties	Applications	References
BNC	Polypyrrole and carbon nanotubes	Regeneration	Thermal stability, mechanical strength, recoverability, swelling ability, electrical conductivity, cytocompatibility	Tissue engineering	Wang et al. (2020b)
	κ-carrageenan	*In-situ*	Mechanical strength, water uptake and retention, swelling, cell viability, and gene expression	Tissue engineering	Cielecka et al. (2018)
	Collagen	*In-situ*	Thermal stability, mechanical strength, cytocompatibility	Wound dressing and tissue engineering	Zhijiang and Guang, (2011)
	Montmorillonite	*Ex-situ*	Antibacterial activity, water release rate, biocompatibility, and wound healing	Biomedical	(Ul-Islam et al., 2013; Sajjad et al., 2019)
	Chitosan	*Ex-situ*	Mechanical strength, water holding capacity and release rate, Cytocompatibility, 3D growth pattern, cell-scaffold interaction	Diagnosis of ovarian cancer	Ul-Islam et al. (2019b)
	Xyloglucan	*—*	Enhanced mechanical properties, biocompatible	Characterize and design biomaterial	Bonilla et al. (2016)
	*—*	*—*	Stripe ordered BC	Scaffolds for nerve, skeleton, and hamstring	Zang et al. (2014)
	Reduced graphene oxide	*—*	Biocompatible, conductive, hydrophilic	Bioelectronics, tissue engineering	Kang et al. (2016)
	NaCl	Regeneration	Porous	Tissue engineering	Khan et al. (2015b)
	CNC and protein	*—*	Biocompatible	Bone tissue engineering	Zhang et al. (2019)
	Graphene oxide	*—*	High tensile strength and biocompatible	Tissue engineering	Si et al. (2014)
	Hydroxyapatite and carboxymethyl cellulose	*Ex-situ*	Crystallinity, thermal stability, cytocompatibility	Biocompatibility	Grande et al. (2009)
	Zinc oxide nanoparticles	Regeneration	Thermal stability, mechanical strength, antibacterial activity, cytocompatibility	Biomedical, bioelectroanalysis	Ul-Islam et al. (2014)
CNCs	PEGDA	3D printing	Mechanical, thermal, and biological properties	Tissue scaffold	NB et al. (2017)
	PVA	Freeze-thaw cycle	Stability, dispersion, and mechanical strength	Tissue scaffold	Shoda and Sugano, (2005)
	PVA	Low-temperature crosslinking	Biocompatibility and mechanical strength	Tissue scaffold	Tummala et al. (2019)
	CNF, alginate	*Ex-situ*	Controlled pore size, biocompatible	Tissue engineering	Al-Sabah et al. (2019)
CNF	Hydroxyapatite	*Ex-situ*	Extra ordinary mechanical properties, biocompatible	Bone tissue engineering	Ao et al. (2017)
	Alginate	3D extrusion printing	3D structure, biocompatible	Cartilage regeneration	Markstedt et al. (2015)
	Alginate	3D printing	Porosity, mechanical strength, crosslinking, biocompatibility	Tissue scaffold	Abouzeid et al. (2018)

### Wound Dressing and Healing

Skin is the largest organ in the human body. It covers the vital organs of the body and serves as a physical and chemical barrier and protective layer against environmental hazards. Skin performs important functions such as detecting sensation, controlling body temperature, regulating the exchange of water and electrolytes, and preventing harmful substances from invading the human body. As the most significant barrier of the human body, skin is often vulnerable to injuries such as high-temperature scald, low-temperature frostbite, mechanical trauma, microbial contamination, and so on (Metcalfe and Ferguson, 2007). Such injuries often lead to the development of wounds at the injury or infection site. If the wound is not healed in time, it may lead to inflammation and other pathological changes. Therefore, to avoid damage to the skin and underlying organs, which could affect the function and cause secondary injuries, it is necessary to treat the wounds effectively and quickly to heal the wounds. One effective approach is to apply wound dressing materials to facilitate the repair process of damaged skin. Wound dressing is an effective method for rapid recovery and treatment of injured tissues. An effective wound dressing material should promote the proliferation and differentiation of wound cells by constructing physical barriers. The materials used as wound dressing should meet the certain criterion: no biological toxicity, no sensitization, good gas permeability, absorption of wound exudates, antimicrobial activity, anti-inflammation, easy removal, and no secondary injury to the wound site (Di et al., 2017).

**FIGURE 4:**
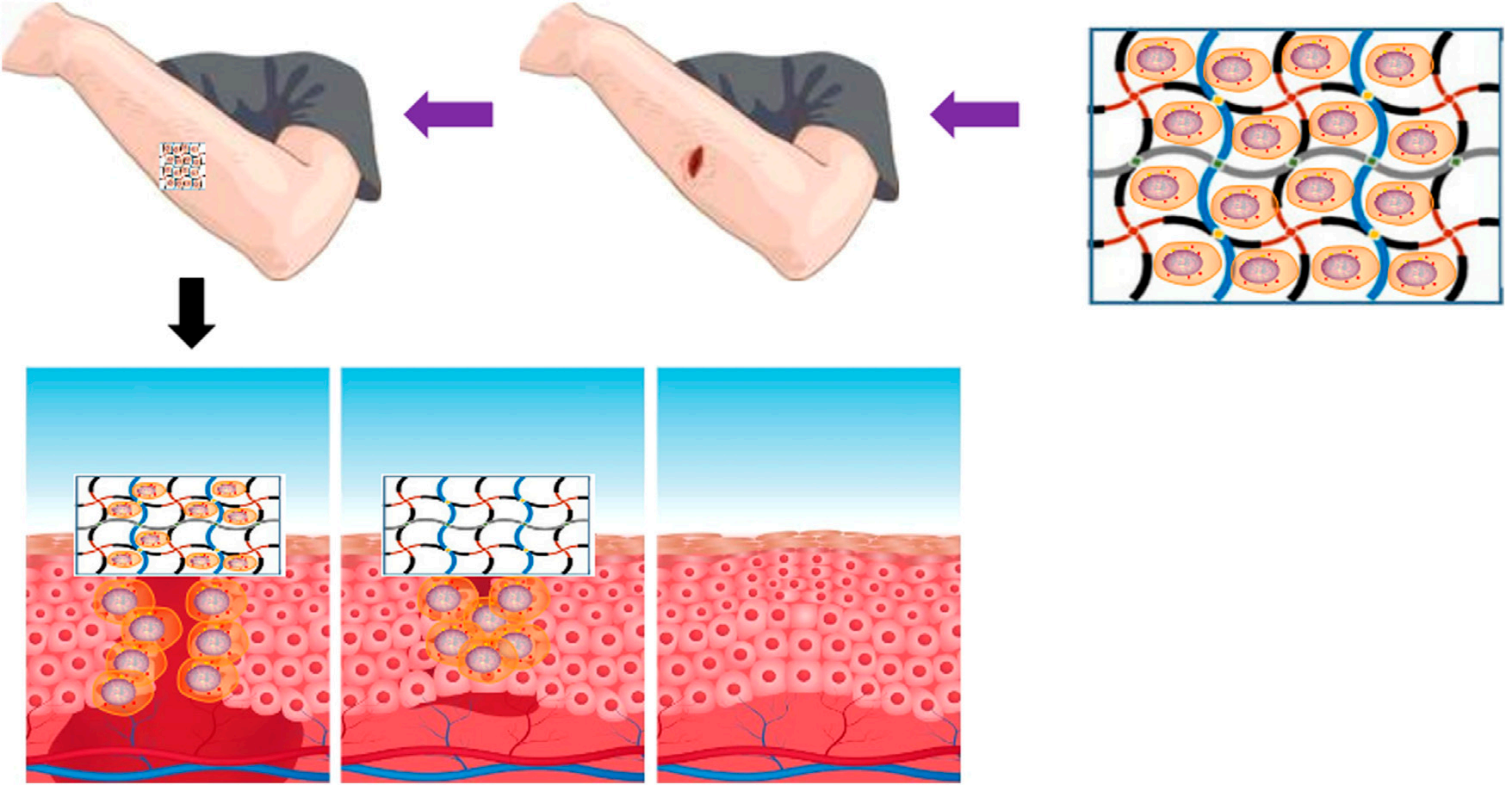
Schematic diagram of wound healing promoting effect of BC hydrogel.

The nanocellulose-hydrogel could be an ideal choice as a wound dressing material because it meets the properties of effective wound dressing materials such as biocompatibility, non-toxicity, and biodegradability (Boateng et al., 2008). The high water content of nanocellulose-based hydrogels provides a humid environment for wounds, while its highly porous and fibrous structure allows gaseous and nutrients exchange. Nanocellulose hydrogels can effectively absorb wound exudates. Among the different types of nanocellulose, the 3D fibrous network of BNC is similar to ECM, thus providing an ideal environment for cell growth and tissue repair. In a study, Loh et al. developed a hydrogel cell carrier based on BNC and acrylic acid BC/AA and utilized it for full-thickness wound healing treatment. The BC/AA composite hydrogel supported the adhesion and growth of human epidermal keratinocytes (EK) and dermal fibroblasts (DF) and quickly transferred the cells from hydrogel to wound. The visual observation, histological analysis, immunohistochemical staining, and transmission electron microscope (TEM) showed good wound closure in the animal model (Loh et al., 2018). In a recent study, Sajjad et al. developed a nanocomposite of BCN and curcumin nanoparticles. The BC/curcumin nanocomposite was utilized as a wound dressing hydrogel that effectively healed the burn wounds in the rat model (Sajjad et al., 2020). A typical wound healing in the skin by using nanocellulose-based hydrogel is demonstrated in Figure 4. In another study, Basu et al. developed calcium cross-linked nanocellulose (NFCs) hydrogel for use as wound dressing material. They developed cation NFC (c-NFC) and carboxylated NFC (a-NFC) hydrogel by modifying the CNFs, and the self-stable c-NFC hydrogel was prepared by adding calcium ions to c-NFC. Similarly, the AC-NFC hydrogel was prepared by mixing c-NFC and a-NFC suspensions at 2:1 ratio by adding calcium ions. The A-NFC and AC-NFC hydrogels showed good water retention ability and maintained a moist environment, highlighting the potential of the physical and chemical properties of the material to promote wound healing. The developed hydrogels did not affect the growth and proliferation of new skin cells on the wound surface as well as their removal as a dressing material did not damage the repaired skin. In addition, the level of reactive oxygen species remained stable, indicating the inert nature of the developed hydrogels. The biocompatible and non-inflammatory properties of the nanocellulose-based hydrogels to skin cells and monocytes further highlight their potential as the ideal wound dressing materials. Table 3 summarizes the development of different nanocellulose-based scaffolds with a variety of materials for wound dressing applications.

**TABLE 3:** The improved properties and wound healing applications of different nanocellulose-based composites.

Cellulose type	Reinforcement material	Synthetic strategy	Improved/added properties	Applications	References
BNC	Collagen	*In-situ*	Thermal stability, mechanical strength, cytocompatibility	Wound dressing and tissue engineering	Zhijiang and Guang, (2011)
	Quaternized chitosan	*Ex-situ*	Yield, porosity, thermal stability, water uptake, cell viability, and antibacterial activity	Antibacterial, biocompatibility	Ao et al. (2020)
	Ag nanoparticles	*In-situ*	Antibacterial activity, biocompatibility, collagen formation, *in vivo* burn wound healing, re-epithelization, and enhanced expression of inflammatory, angiogenesis, and growth factor genes	Third-degree burn wound healing	Jiji et al. (2020)
	Oxidized BC, chitosan, collagen	*Ex-situ*	Mechanical strength, antimicrobial activity, cell viability, *in vivo* and *in vivo* biodegradation, and hemostasis	Hemostasis and wound healing	Yuan et al. (2020)
	BC, chitosan, and diamond nanoparticles	*Ex-situ*	Enhanced electric modulus, biocompatible	Wound dressing	Ostadhossein et al. (2015)
	Poly (2-hydroxyethyl methacrylate) and silver nanoparticle	*Ex-situ*	Mechanical strength, optical transparency, antibacterial activity, cytocompatibility	Transparent wound dressing	Di et al. (2017)
	ZnO	*Ex-situ*	Biocompatible, Antimicrobial	Wound dressing	Janpetch et al. (2016)
	MTT	*Ex-situ*	Antimicrobial	Wound healing	Ul-Islam et al. (2013)
	Vaccarin	*In-situ*	Biocompatible	Wound dressing	Qiu et al. (2016)
	Curcumin	*Ex-situ*	Crystallinity, reepithelization, vascularization, wound closure, antibacterial activity	Partial-thickness skin burn	Sajjad et al. (2020)
	Zinc oxide nanoparticles	Regeneration	Thermal stability, mechanical strength, antibacterial activity, cytocompatibility	Antibacterial	Ul-Islam, Khattak, et al. (2014)
	Titanium dioxide nanoparticles	*In-situ* and regeneration	Antibacterial activity, biocompatibility	Antibacterial	(Khan et al., 2015a; Ullah et al., 2016a)
CNC	Carboxymethyl chitosan	High-pressure homogenizer	Self-healing, biocompatibility	Deep partial-thickness skin burns	Huang et al. (2018)
	Ag	*Ex-situ*	Antimicrobial	Antimicrobial wound healing	Luzi et al. (2019)
CNF	Hydroxypropyl trimethyl	TEMPO oxidation	Metal cation crosslinking	Wound dressing	Arfin, (2020)
	Chitosan and PVA	*Ex-situ*	Antibacterial	Wound healing	Poonguzhali et al. (2018)
	Gelatin and Ag-NH_2_ nanoparticles	TEMPO oxidation method and high-pressure homogenization	Mechanical strength, self-recovery, antibacterial activity, hemostatic performance	Wound dressing	Liu et al. (2018)
	Poly [2-(methacryloyloxy) ethyl] trimethylammonium chloride	Crosslinking	Nanofibrous and porous structures	Treatment of *Candida albicans* infections	Vilela et al. (2019)

### Drug Delivery

A drug delivery system refers to a mechanism of transporting a drug or a therapeutic agent to the target site to achieve its maximum therapeutic effect for treating a disease or illness. In general, drugs are delivered to the target site by using suitable carriers. An ideal carrier used for delivery of drug to the target site must demonstrate some unique features or offer advantages like biocompatibility, low or no toxicity, high drug loading or encapsulation efficiency, targeted and efficient drug transport to the target site, simple preparation, and low production cost, extended *in vivo* circulation time, and so on (Li et al., 2021). Compared to the traditional drug delivery systems, the hydrogel-based drug delivery systems are less toxic and have minimal side effects (Ribeiro et al., 2014).

For its use in developing drug delivery systems, nanocellulose possesses all important features such as high hydrophilicity, stability, biocompatibility, non-toxicity, appropriate mechanical strength, biodegradability, easy surface modification, and low preparation cost. Furthermore, due to its 3D fibrous network, porosity, and high surface area, nanocellulose-based hydrogels can offer high drug loading capacity while flexibility in its unique surface chemistry allows the controlled release. In a study, Poonguzhali prepared the composite membrane of ampicillin-loaded sodium alginate and CNC through the solution casting method. The sodium alginate/CNC composite membrane showed the release of ampicillin showed greater drug release compared to the membrane without CNC. The developed composite showed extended drug release for up to 500 min and demonstrated good swelling behavior in an alkaline medium, indicating that it could be used for extended drug release (Poonguzhali et al., 2018). In another study, Hong et al. carboxymethylated CNF and prepared the ciprofloxacin-montmorillonite (CIPMMT) composite for sustained release of antibiotic drugs. Herein, carboxymethylated cellulose nanofibrils (CMCNF) were used as the carrier. Although montmorillonite can delay the sustained release of drugs, the delay time is only 6–24 h at most. The CMCNF-CIP-MMT complex drug system was prepared by adding 1.5, 2, and 3% CMC-CNF into the CIP-MMT system. The *in vitro* release experiments showed sustained drug release by the CIP-MMT system. The sustained release of CIP for more than 6 h and the dissolution of the matrix can be delayed by adding CMCNF into the system. The sustained release process of the drug increases with the increase of CMCNF, and the sustained release of 3% CMCNF-CIP-MMT can last for more than 48 h. The antibacterial experiment showed that the 3% CMCNF-CIP-MMT composite showed stable antibacterial activity within 12 days (Hong et al., 2019). In another study, Jagadeesen et al. used CNCs extracted from rice husk as raw materials. They further modified the CNCs by imparting magnetic property (m-CNCs) through the co-precipitation method. The m-CNCs were dispersed in alginate-based hydrogel beads, which enhanced the mechanical strength and regulated the drug release behavior. It was found that the existence of m-CNCs not only improved the magnetic properties of alginate hydrogel beads but also enhanced the stability and swelling rate of hydrogel beads. The developed hydrogel showed high loading of ibuprofen and controlled release for extended time period (Supramaniam et al., 2018).

**FIGURE 5:**
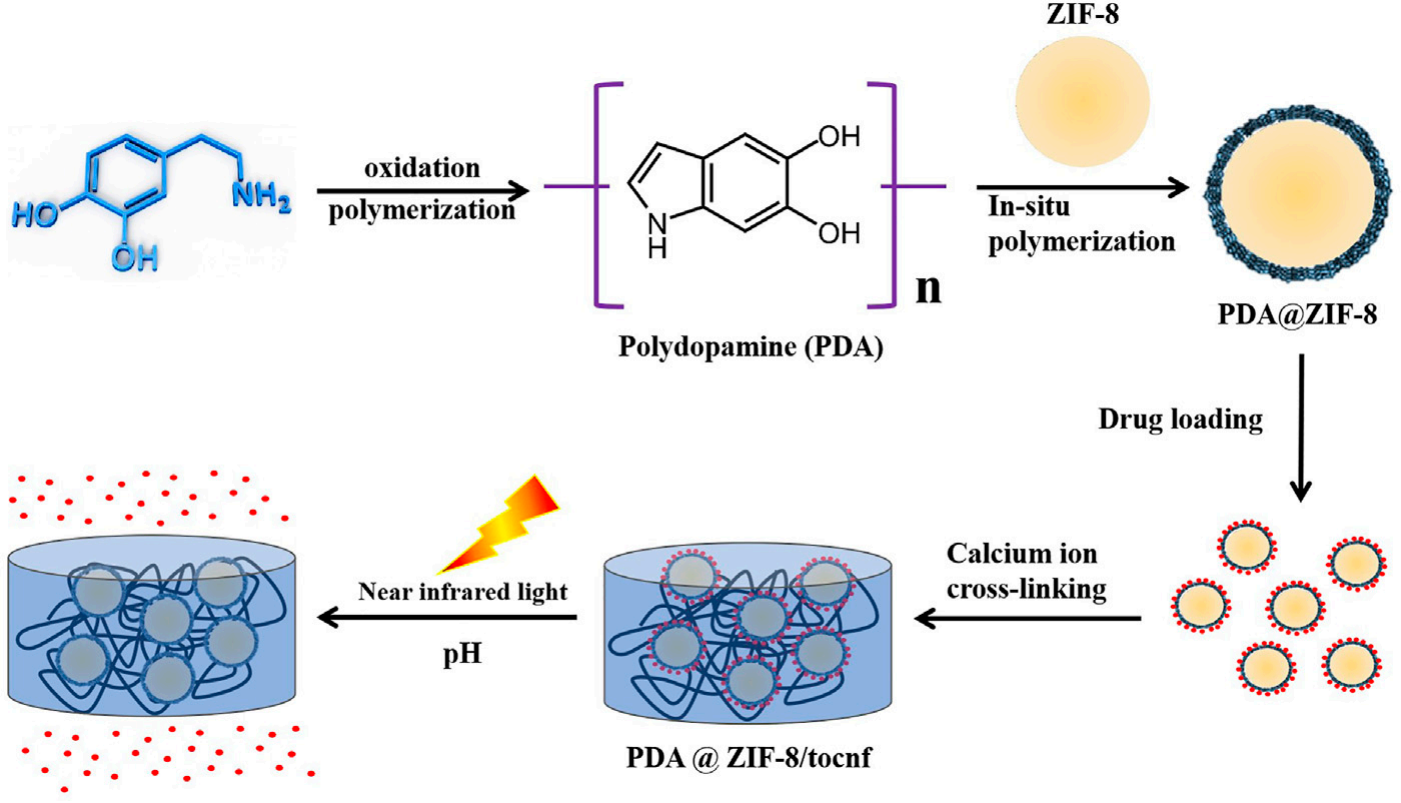
Preparation of composite hydrogel and its application in drug delivery.

In order to enhance the stability of drug binding and ensure the controlled release of drugs, the nanocellulose-hydrogel drug carrier system can be chemically modified to prepare the stimulus-responsive drug carriers, such as pH, temperature, light, and ultrasonic response (Karimian et al., 2019). The stimuli-responsive hydrogels are used as the drug carrier for constructing drug controlled release system, which could potentially solve the issue of administration of multiple drugs in traditional administration mode. In addition, such hydrogels could reduce the stimulation effect of drugs on the normal cells and ensure controlled and targeted delivery of drugs, which could ultimately lead to the improved therapeutic effect of the drug. In recent years, stimuli-responsive hydrogels based on nanocellulose have received immense consideration. In a study, Li et al. developed a sandwich structure of BNC with polyaniline through chemical oxidation polymerization. Herein, polyaniline was densely arrayed along the cellulose fibers, and a model drug berberine hydrochloride was diffused within the hydrogel matrix. The sandwich structure allowed a controlled release under the effect of varying pH and electric fields. The system showed fast drug release in an alkaline environment and slow in an acidic environment (Li et al., 2018). In another study, Yunessnia et al. developed a CNCs/chitosan nanocomposite hydrogel. With the increasing concentration of chitosan, the isoelectric point and swelling ratio of the composite hydrogel increased. The CNC/chitosan showed *in vitro* release of theophylline, where the cumulative drug release at pH 1.5 was significantly higher than at pH 7.4. A cumulative release of 85% was achieved at pH 1.5 when 3% chitosan was used. The findings of this study indicate that the CNC/chitosan nanocomposite could be used for gastric-specific drug delivery (Yunessnia lehi et al., 2019). In a more detailed study, Liu et al. developed a nanocellulose hydrogel-based drug carrier with multiple response characteristics (pH, near-infrared light, and long-term slow-release). *In-situ* growth of nano zinc-based MOF(ZIF-8) was controlled by nano dopamine (PDA) as a template to obtain PDA@ZIF-8 nanocomposites. Then, the drug tetracycline hydrochloride was loaded by PDA@ZIF-8 nanocomposites, and then the PDA@ZIF-8/TOCNFs composite hydrogel material with drug slow-release function was prepared by calcium ion crosslinking. The results showed a maximum encapsulation efficiency and loading rate of tetracycline hydrochloride by PDA@ZIF-8. In addition, PDA@ZIF-8/TOCNFs composite hydrogel has good pH and near-infrared light-sensitive drug release characteristics. In the acidic buffer system, the sustained release time of the drug was as long as 85 h, and the drug release rate was 72%, and there was no abrupt release phenomenon at the initial stage. These results show that the PDA@ZIF-8/TOCNFs composite hydrogel has good drug release characteristics. Furthermore, the PDA@ZIF-8/TOCNFs composite hydrogel did not show toxicity to human umbilical vein cells, thus indicating their biocompatible nature (Khamrai et al., 2019). A schematic illustration of the preparation of composite hydrogel and its application in drug delivery is shown in Figure 5. Arash et al. developed CNF/chitosan composite as a drug carrier for preparing a pH-responsive drug delivery system for treating trichomoniasis. The chitosan nanocapsules were prepared by embedding chitosan polymer on magnetic nanoparticles as the template. The nanocapsules were doped with CNF by tannic acid as nanochips for the delivery of drug to the target sites. The developed CNF/chitosan system was biocompatible and demonstrated pH-response drug release (Yunessnia lehi et al., 2019). Table 4 summarizes the development of different nanocellulose-based scaffolds with a variety of materials for drug delivery applications.

**TABLE 4:** The improved properties and drug delivery applications of different nanocellulose-based composites.

Cellulose type	Reinforcement material	Synthetic strategy	Improved/added properties	Applications	References
BNC	Graphene oxide and ibuprofen	*Ex-situ*	Cell viability, sustained drug release *in vitro*	Drug delivery	Luo et al. (2017)
	Oxidized BC and polyethyleneimine	*Ex-situ*	Adsorption, sustained drug release *in vitro*, biocompatibility	Drug delivery	Chen et al. (2018b)
	Polyaniline	*Ex-situ*	pH-responsiveness, electrical conductivity	Controlled drug delivery	Li et al. (2018)
	—	Freeze-drying	pH-dependent release rate, 3D structure	Controlled drug delivery	Huang et al. (2013)
CNCs	CMC, starch, and pectin	*Ex-situ*	pH resistance	Probiotics delivery	Khorasani and Shojaosadati, (2017)
	Chitosan	*Ex-situ* TEMPO oxidation method	Ionic crosslinking, extended drug release	Drug delivery	Maestri. et al. (2020)
	Chitosan	*Ex-situ*	pH-sensitivity, high swelling	Drug delivery	(Q et al., 2019)
	Alginate	High-pressure homogenization	Magnetic behavior, high swelling, stability	Controlled drug delivery	Supramaniam et al. (2018)
CNFs	Chitosan	TEMPO oxidation method	High swelling, crosslinking stability	Drug delivery	(Q et al., 2019)
	Quaternary ammonium salt group	High-pressure homogenizer	High swelling, high drug loading, and controlled release	—	Hujaya et al. (2018)
	PANIPAM	Free radical polymerization	High swelling, compression strength, dual responsive	Drug delivery	Masruchin et al. (2017)

### Synthetic Organs

#### Bone and Cartilage

Bone is a hard, dynamic, and complex tissue. Although it has the ability to regenerate in case of small damage or crack; however, a major injury may lead to severe bone damage that requires the introduction of a graft. The non-toxicity and good biocompatibility of certain minerals such as hydroxyapatite (Hap), silica, calcium carbonate and chloride, and nanoclay have led to their use in biomedical applications. In addition, nanoclays are known as good mechanical reinforcement agents. Hap, which is a phosphate mineral comprising calcium phosphate and is otherwise known as bone mineral, has been used in bone grafting (Ullah et al., 2020) and for bone drug delivery (Ullah I. et al., 2019).

A number of reports have shown impregnation of the cellulose matrix with Hap for bone regeneration applications (Wang et al., 2011; Luo et al., 2014; Ramani and Sastry, 2014). For instance, Fang et al. utilized phosphorylated BNC to induce the formation of calcium phosphate in the BNC matrix; the average pore diameter of the BNC-Hap composites was approximately 1 µm while that of the control BNC was around 100 nm. Moreover, the BNC-Hap composites showed better osteoblast proliferation and mesenchymal stem cell differentiation, although no external differentiation markers were supplied (Fang et al., 2009). The synthesis of BC composites with calcium-deficient hydroxyapatite (BNC-CdHap) has also been reported for bone regeneration applications. The authors reported enhanced osteoprogenitor cell adhesion on the BNC-CdHap surface, indicating that these composite scaffolds are suitable for bone regeneration applications and suggested further investigation (Zimmermann et al., 2011). Grande et al. developed BNC-Hap nanocomposites through an *in-situ* impregnation method (Grande et al., 2009). The nanocomposites were found suitable for biomedical applications. Wan et al. phosphorylated BNC to enhance its ability to induce Hap production, for which it was better than pristine BNC (Wan et al., 2007). Hutchens et al. fabricated BNC-Hap nanocomposites mimicking the biomineralization of natural bone (Hutchens et al., 2006). Silica, the major constituent of sand, is obtained through quartz purification and sand mining. Yano et al. synthesized BC-silica composites through both *in-situ* and *ex-situ* impregnation methods (Yano et al., 2008). Maeda et al. fabricated BC-silica composites *via* an *ex-situ* method followed by hot pressing; the composites showed high tensile strength (185 MPa) and Young’s modulus (17 GPa) (Maeda et al., 2006). BC-silica composites have also been proposed as aerogels (Cai et al., 2012), light-emitting materials (Barud et al., 2008; Cai et al., 2012), and adsorbents for water purification (Sai et al., 2013). Mineralized tissues also contain calcium carbonate (CaCO_3_) (Stoica-Guzun et al., 2012), and BNC-CaCO_3_ composites have been synthesized (Serafica et al., 2002; Shi et al., 2009). Stoica-Guzun et al. studied the effect of CaCO_3_ on BNC pellicles using sodium carbonate (NaCO_3_) and calcium chloride (CaCl_2_) as reactants; bigger crystals of BNC-CaCO_3_ composites with different shapes were obtained in ultrasound-irradiated samples compared to non-irradiated samples (Stoica-Guzun et al., 2012). They also studied the effect of microwave irradiation using the same reactants and found diverse polymorphism and substantial morphological differences for irradiated BNC-CaCO_3_ crystals were found suitable for medical and industrial applications (Stoica-Guzun et al., 2013). The surface chemistry and physical features have been further improved by developing BNC composites with calcium phosphates (de Olyveira et al., 2017). Saska et al. impregnated BC with calcium chloride (CaCl_2_) and sodium hydrogen phosphate (Na_2_HPO_4_); the obtained composites were effective for defect regeneration in rat tibial bones with complete bone regeneration within 4 weeks of implantation without any inflammation (Saska et al., 2011). Researchers have shown that polymer nanoclay composites show good thermal and mechanical stability, biodegradability, and barrier properties (Algar et al., 2016). Perotti et al. fabricated BNC-laponite clay nanocomposites with different BNC to clay ratios through *ex-situ* penetration from a water suspension of the clay. The obtained nanocomposites were uniform and stable with good tensile strength and Young’s modulus (Perotti et al., 2011). Ul-Islam et al. synthesized BNC-montmorillonite (MMT) composites with different MMT concentrations. When testing their antimicrobial property against *Staphylococcus aureus* and *Escherichia coli*, they found better antibacterial properties with higher MMT concentrations (Ul-Islam, Khan, et al., 2013).

In addition to BNC, Kapender et al. prepared an injectable biological hydrogel comprised of nanocellulose and PVA. Herein, the nanocellulose reduced the surface features of PVA hydrogel and improved the internal structural stability of the composite hydrogel. The developed composite hydrogel demonstrated enhanced physiological properties and remained stable at ambient temperature. Importantly, the increasing concentration up to a certain limit enhanced the injectability of the hydrogel. The nanocellulose/PVA scaffold supported the growth of osteoblasts *in vitro* and regenerated the damaged bone tissues *in vivo*. Most importantly, the scaffold showed *in vivo* degradation at a slow rate, thus prevents the second surgery for removal of scaffolds and enhanced the bone recovery rate (Phogat et al., 2020). The outcomes of the study show that nanocellulose-based scaffolds could be a suitable candidate for developing tissue engineering scaffolds. In another study, Huang et al. developed a porous and lightweight scaffold for bone tissue engineering application by *in-situ* coating the CNCs matrix with Hap. The mechanical strength and water stability of the scaffold were improved by cross-linking with polymethyl vinyl ether malonic acid (PMVEMA) and polyethylene glycol. Furthermore, the scaffold demonstrated high *in vitro* biocompatibility and stabilized the bovine serum albumin, thus demonstrated their potential for bone tissue engineering applications (Huang et al., 2018).

#### Neural Tissues

Neurons represent the longest cells in the body that perform important functions in the body, such as receiving sensory signals from the external environment and sending commands to muscles. These have limited regeneration ability; nevertheless, the neuronal stem cells can self-renew and differentiate into neurons under appropriate growth conditions when cultured on a scaffold. An earlier study reported the differentiation of neural stem cells on the surface of a stiff surface of a biological scaffold (Leipzig and Shoichet, 2009).

The damage to neural tissues can be restored by developing neural implants, which are synthetic devices capable of stimulating parts and structure of the nervous system through electrical circuitry or electrical activity of the neurons. The conductive composite materials, such as those containing polymers and nanoparticles, could be used in the development of neural implants due to their conductive nature and biocompatibility. In another study, nanocarbon/cellulose hybrid hydrogel was prepared through *in-situ* modification of BNC by using the amphiphilic comb-like polymer (APCLP) as a stabilizer. The developed hydrogel demonstrated enhanced neuronal bilayer formation (Kim et al., 2017). In another study, Kuzmenko et al. formulated an ink comprised of CNF and carbon nanotubes that was printed into 3D scaffolds. The printed scaffolds supported the adhesion, growth, proliferation, infiltration, and differentiation of neurons (Kuzmenko et al., 2018). Compared to the conventionally prepared nanocellulose-based scaffold, the 3D scaffolds could be a better choice for treating various neural damages.

#### Cardiovascular Tissues

The cardiovascular system, also known as the blood circulatory system, is a complex system comprised of heart, heart valves, and blood vessels (arteries, capillaries, and veins). Any damage or irregularity to these organs leads to the development of different cardiovascular diseases. Some of these diseases could be treated by implanting synthetic drafts. The materials used in the development of synthetic drafts or implants must demonstrate certain features such as oxygen uptake, nitric oxide production, response to shear stress, anticoagulation, and biocompatibility (Wang D. et al., 2020). Due to the biocompatible and immunocompatible nature of nanocellulose, it can be used in the development of different cardiovascular devices such as artificial blood vessels, heart valves, aorta, prostheses, and others. In an earlier study, Millon and Wan developed CNF/PVA nanocomposite that demonstrated high mechanical strength comparable to the heart valve and aorta (Millon and Wan, 2006). In another study, a BNC-based tubular structure was developed that supported the adhesion and proliferation of human umbilical vein endothelial cells (HUVECs), smooth muscle cells (SMCs), and fibroblasts, thus could be ideal for developing artificial blood vessels (Zang et al., 2015). To date, several nanocellulose-based products such as BASYC^®^, Securian^®^, and SyntheCel^®^ have been commercialized for their use as cardiovascular implants (Ludwicka et al., 2016).

#### Cornea

The eye is a complex organ in the body. In the eye, the eyelids, conjunctiva, and tear glands protect the cornea from injury and maintain its transparency. Any damage to the cornea can be treated by using implants, which should possess high mechanical strength, optical transparency, biocompatibility, permeability to oxygen, and support the epithelization (Wang J. et al., 2010). In an earlier study, a nanocellulose-based scaffold supported the adhesion and proliferation of corneal stromal cells (Jia et al., 2009). In another study, Tummala et al. developed composite hydrogel based on PVA and CNCs. The developed composite demonstrated macroporous 3D network structure and high optical transparency. Furthermore, the scaffold supported the *in vitro* growth of human corneal epithelial cells (HCE-2 cells) and showed a high affinity for protein. The findings of this study demonstrate the potential of CNC/PVA scaffold for their use in the development of ophthalmic applications (Tummala et al., 2019); however, it warrants further investigation regarding the *in vivo* toxicity and stability analyses prior to clinical and commercial use.

#### Dental Implants

Dental implants are the mechanical anchors placed in the mandible which allow the adhesion and growth of cells and tissues around the grooves. These further allow the remodeling of bone around the implant. The materials used in the development of dental implants must demonstrate certain features such as high mechanical strength, porosity, biocompatibility, and non-toxicity. The success of implanting material depends on its overloading, surface features and micro-gap, abutment connection, material type, bone quality, and implant position and geometry (Nimbalkar et al., 2020). Different types of nanocellulose have shown promising results for their use in the development of dental implants. An earlier study developed a BNC-based composite with sodium alginate as a dressing material for surgical wounds in the oral mucosa (Chiaoprakobkij et al., 2011). Similarly, the cellulose whiskers with commercial mineral trioxide aggregate as a reinforcement material showed accelerated hardening (Jinga et al., 2014). The outcomes of these studies demonstrate the potential of nanocellulose in the development of dental implants.

Table 5 summarizes the development of different nanocellulose-based scaffolds with a variety of materials for the development of different synthetic organs.

**TABLE 5:** The improved properties and development of synthetic organs prepared from nanocellulose-based composites.

Cellulose type and reinforcement material	Synthetic strategy	Improved/added properties	Applications	References
Gelatin, hydroxyapatite, and procyanidins	*Ex-situ*	Porosity, mechanical strength, cell viability, *in vivo* bone formation	Bone tissue engineering	Huang et al. (2017)
Hydroxyapatite, CNC	—	Improved thermal properties, biocompatible	Bone tissue engineering	Niamsap et al. (2019)
Hydroxyapatite and graphene oxide	*Ex-situ*	Water uptake, *in vitro* degradation, cell adhesion and growth, and ALP activity	Bone tissue engineering	Ramani and Sastry, (2014)
HAp	Post-synthesis loading	Ca^2+^ and PO_4_^2−^ present, significant improvement of osteoblast growth, adhesion, and osteoconductivity on BC-HAp membranes		Tazi et al. (2012)
BNC	3D printing	Biocompatible and suitable mechanical properties	Artificial kidney and liver	Recouvreux et al. (2011)
BNC	*In-situ*	Bilayer, mechanical stability, porous	Neo cartilage formation	Martínez Ávila et al. (2015)
BNC and agarose	Molding	Aligned fiber	Neural cell proliferation	Zang et al. (2014)
BNC	*In-situ*	Tubular structure, biocompatible	Blood vessels	Lin et al. (2013)
BNC and polydimethylsiloxane	Molding	Tubular and biocompatible	Artificial blood vessels	Zang et al. (2015)
BNC	Freeze-drying	Biocompatible, transparent, and suitable mechanical properties	Artificial cornea	Jia et al. (2009)
GO	*Ex-situ*	Less crystalline	Neural cell proliferation	Kim et al. (2017)
Fibrin	*Ex-situ*	Biocompatible	Vascular graft	Karimian et al. (2019)
Peptide	Crosslinking	Biocompatible	Blood vessels	Lin et al. (2013)
BNC and PVA	*Ex-situ*	Suitable mechanical properties and anisotropic behavior	Heart valve	Mohammadi, (2011)
BNC and PVA	*Ex-situ*	Transparent, UV absorbent, high mechanical strength, and thermal stability	Artificial cornea	Wang et al. (2010a)
CNC and ionic liquids	Regeneration	Transparent, high WHC	Ocular bandage	Patchan et al. (2013)
BNC, otoliths, and collagen	Post-synthesis loading	Formation of bone tissue with higher osteoblast activity, high degree of regularity, and osteo-reabsorption activities	Bone tissue engineering	Olyveira et al. (2011)
BNC and Col_1_	Post-synthesis cross-linking	Tensile strength and elastic modulus for BC-Col_1_ decreased, a slight increase in strain at break, similar cell morphology, and cell proliferation/viability	Bone tissue engineering	Saska et al. (2012)

### 3D Bioprinting

Currently, researchers are paying great attention to tissue engineering using advanced technologies such as 3D bioprinting (Aljohani et al., 2018; McCarthy et al., 2019b, 2019a). The successful proliferation of animal cells on biopolymers has led to the development of three-dimensional (3D) scaffolds for *in vivo* tissue engineering and regenerative medicine applications. An ideal 3D scaffold must be biocompatible and nontoxic and must possess appropriate surface chemistry to support the adhesion, proliferation, and differentiation of cells, as well as resemble the micro-scale morphology of ECM (Kim and Kim, 2015). Moreover, the 3D scaffold needs to have uniform and interconnected pores to facilitate the cell infiltration, vascularization, and nutrients and waste exchange, besides possessing suitable mechanical strength to assist the tissue formation without breakage (Banerjee and Park, 2015; Wu et al., 2015). To date, cellulose has been little evaluated for its ability to form bioink for 3D printing due to its high rheology and stable structure that makes it hard to dissolve in common solvents. However, the potential advantages of using nanocellulose in the development of 3D printed scaffolds are quite clear, and some earlier success reports are available. For example, Recouvreux et al. synthesized an organ-like 3D hydrogel of BC and characterized its structural features, mechanical strength, and biocompatibility; they believe it has potential as an implantable tissue and organ scaffold for organs such as the kidney or liver (Recouvreux et al., 2011). In another study, de Souza, Olival-Costa et al. implanted a BC membrane in rabbits and investigated its response in terms of medialization, inflammation, and healing of the vocal folds; the BC membrane remained stable for 120 days and did not demonstrate any major drawbacks, indicating its suitability for the medial displacement of the vocal folds (De Souza et al., 2011). In another study, a BNC-based ear-shaped model was created from the reconstruction of gradient-echo magnetic resonance imaging. For this purpose, Nimeskern et al. bioprinted BNC using a negative silicone mold by manipulating the bacterial activity to reproduce the large-scale features of the outer ear to generate patient-specific ear shapes (Nimeskern et al., 2013). In another study, a conductive ink comprised of CNF and CNTs was formulated and printed under optimized conditions of pH-dependent surface charges. The 3D printed scaffold supported the adhesion, proliferation, and differentiation of neuronal cells (Kuzmenko et al., 2018). These advancements demonstrate the potential of nanocellulose as a promising material in tissue engineering for the development of a wide range of materials, such as for treating spinal cord injury (Bedir et al., 2020).

Table 6 summarizes the development of different 3D printed nanocellulose-based scaffolds for biomedical applications.

**TABLE 6:** The improved properties, 3D printing, and biomedical applications of different nanocellulose-based composites.

Cellulose type	Reinforcement material	Synthetic strategy	Improved/added properties	Applications	References
CNF	Alginate	3D extrusion printing	3D structure, biocompatible	Cartilage regeneration	Markstedt et al. (2015)
CNCs	PEGDA	3D printing	Mechanical, thermal, and biological properties	Tissue scaffold	NB et al. (2017)
BNC	—	3D printing	Biocompatible and suitable mechanical properties	Artificial kidney and liver	Recouvreux et al. (2011)
CNFs	Alginate	3D printing	Porosity, mechanical strength, crosslinking, biocompatibility	Tissue scaffold	Abouzeid et al. (2018)

### Biosensing

Biosensing is a mechanism of detecting a biomolecule, a biological structure, or a microorganism by using a biosensor. A biosensor is an analytical device that transforms the biological response into an output signal by using the biorecognition elements such as enzymes, antibodies, nucleic acids, and bacteriophages (Farooq et al., 2018). Nanocellulose, owing to its unique surface chemistry, high surface area, high aspect ratio, flexibility, mechanical strength, and biocompatibility, could be an ideal material for the development of biosensors. Although nanocellulose is non-conductive, its composites with conductive polymers and nanoparticles demonstrate electrical conductivity and thus could be used in the development of biosensors. In a study, Jasim et al. modified the fibers in BNC with polyaniline through oxidative polymerization and further impregnated the modified fibers with single-walled carbon nanotubes. The developed composite showed high electrical conductivity and thus could be used in the development of a biosensor. Abdi et al. developed an electrochemical biosensor by combining CNC, polyaniline, and ionic liquid on a modified screen-printed electrode. Herein, cholesterol oxidase was used as the biorecognition element for the detection of cholesterol level. The developed biosensor showed operational repeatability, low limit of detection, and high sensitivity (Abdi et al., 2019). In another study, Farooq et al. developed an electrochemical biosensor based on BNC by using a phage as the biorecognition element. They first carboxylated the BNC and then modified it with polyethyleneimine (PEI) to allow an electrostatic interaction between the negatively charged phages and positively charged BNC/PEI matrix. The modified BNC/PEI matrix was impregnated with carbon nanotubes. The modified matrix allowed the immobilization of phages. The developed sensor effectively detected *S. aureus* and differentiated the live and dead cells (Farooq et al., 2020). Nanocellulose-based sensors have also been developed for the detection of glucose level in the blood (Wang W. et al., 2010). These nanocellulose-based sensors offer several advantages like low cost, reproducibility, stability, and biocompatibility. Table 7 summarizes the development of different nanocellulose-based scaffolds with a variety of materials for biosensing applications.

**TABLE 7:** The improved properties and biosensing applications of different nanocellulose-based composites.

Reinforcement material	Synthetic strategy	Improved/added properties	Applications	References
Poly aniline and carbon nanotubes	*Ex-situ*	Thermal stability, electrical conductivity	Biosensors, solar cells, bio-electronic devices	Jasim et al. (2017)
Carbon nanotubes and poly (ethylene imine)	*Ex-situ*	Sites for phage immobilization, Mechanical strength, conductivity, antibacterial activity, stability of the sensor	Biosensing	Farooq et al. (2020)
CNC and GO	*Ex-situ*	Proximity sensing ability	Optoelectronic sensing devices	Sadasivuni et al. (2015)
CNC and GO	*Ex-situ*	Flexible, transparent, conductive	Biofluid separation	Xiong et al. (2016)

## Conclusion and Future Perspective

As a biodegradable, non-toxic, and renewable material with appropriate mechanical strength, nanocellulose-based hydrogels have been proved to be a promising material for tissue engineering and regenerative medicine applications. In this review, the preparation methods of nanocellulose-based hydrogels are introduced, and their applications in biomedicine and tissue engineering are summarized, especially in developing tissue engineering scaffolds, wound dressings, drug carriers, synthetic organs, biosensing materials, and 3D printed scaffolds. However, there are several problems in the application of nanocellulose-based hydrogels in bioengineering in the research process.

First, the long-term biosafety of hydrogels made by CNCs and CNFs in biomedicine has not been evaluated systematically. Although natural cellulose such as BNC is biocompatible and non-toxic, most of these studies are based on cell or histopathological experiments or short-term examinations. For example, when the nanocellulose-based hydrogel is applied to drug delivery systems, the long-term effects of these drug carriers, including the long-term toxicity, biocompatibility, immunogenicity, pharmacokinetics, and pharmacodynamics, should be verified in animal models before the clinical transformation. Secondly, the nanocellulose-based hydrogels are reported to be a high-quality tissue engineering scaffold for direct application in humans; however, these lack innate antibacterial, antioxidant, and regenerative activity, thus the nanocellulose-based biomaterials need the impregnation or doping of antibiotics or additives to enhance their biological activity such as for tissue regeneration and preventing the infection properly. Third, it is often difficult to load drugs or cells into the hydrogels for controlled drug delivery, which requires further design of hydrogels. To ensure the biological safety of hydrogel during drug delivery, attention should be paid to its preparation, such as by developing innoxious solvent, green synthesis method, and the non-toxic crosslinking agent.

The optimization of the synthesis process, post-synthesis processing, and improving the physical and chemical modification methods of nanocellulose-based hydrogels can expand their applications to new areas and reach the stage of commercialization. For instance, the development of effective wound dressings, controlled and sustained-release drug delivery systems, and developing stimulus-response types sensitive to pH, temperature, humidity, infection, etc., can have potential clinical applications.

## Data Availability

The original contributions presented in the study are included in the article/Supplementary Material, further inquiries can be directed to the corresponding authors.
